# Use of Serotonergic Drugs in Canada for Gastrointestinal Motility Disorders: Results of a Retrospective Cohort Study

**DOI:** 10.1155/2016/5797804

**Published:** 2016-05-30

**Authors:** Biniam Kidane, Farouq Manji, Jennifer Lam, Brian M. Taylor

**Affiliations:** Schulich School of Medicine & Dentistry, University Hospital, Western University, Rm C8-114, 339 Windermere Road, London, ON, Canada N6A 5A5

## Abstract

*Background.* Surgery for GI dysmotility is limited to those with severe refractory disease. Though effective, use of serotonergic promotility drugs has been restricted in Canada due to adverse events. We aimed to investigate utilization of promotility serotonergic drugs in patients under consideration for surgical management.* Methods.* A retrospective cohort study was conducted using prospectively collected data. The study population included consecutive patients referred to a motility clinic for consideration of bowel resection at a Canadian tertiary hospital (1996–2011). Univariable tests and multivariable logistic regression analyses were used to assess predictors of serotonergic drug use.* Results.* Of 128 patients, the majority (*n* = 98, 76.6%) had constipation-dominant symptoms. Only 25% (*n* = 32) had tried serotonergic promotility drugs. There was no association between use of these drugs and severity of constipation nor was there an association between serotonergic drug use and presence of diffuse dysmotility (all *p* > 0.05). The majority of patients (*n* = 97, 75.8%) underwent some type of surgical resection, which was associated with considerable morbidity (*n* = 13, 13.4%).* Conclusions.* Surgical management of GI dysmotility results in serious morbidity. Serotonergic promotility drugs may allow patients to avoid surgery but disease severity does not predict use of these drugs.

## 1. Introduction

Functional gastrointestinal disorders are prevalent illnesses that result in low quality of life and high health resource utilization. The estimated annual health care costs of these disorders in the US exceed $19.2 billion [1–3]. Functional gastrointestinal disorders (FGIDs) are heterogeneous and have been classified according to Rome III criteria. Although heterogeneous, the unifying theme is disordered motility [4].

Although operative management is generally avoided, a subset of patients have such significant disease that bowel surgery is used as a last resort [5]. Few patients undergo surgical bowel resection, given the potential for significant postsurgical morbidity [6]. Serotonergic drugs have been used for the treatment of FGIDs. Cisapride was an early and widely used drug. Its prokinetic effects throughout the GI tract made it suitable for a wide range of GI motility disorders. In 2000 however the drug was withdrawn due to concern over adverse cardiovascular events [7]. Similarly, Tegaserod was a serotonergic drug initially introduced for constipation-dominant irritable bowel syndrome (IBS), but withdrawn in 2007 in the US and Canada due to reports of cardiovascular adverse events in postmarketing studies [8, 9]. These drugs provided an alternative to standard medical therapy (i.e., lifestyle modification and laxative use) before surgical intervention. Due to their withdrawal there has been a paucity of efficacious promotility drugs available for the treatment of GI motility disorders in Canada.

Patients are referred to our GI motility clinic with disorders ranging from slow transit constipation to constipation-dominant IBS. These patients have such significant and refractory diseases that they are often managed by multiple physicians before referral to our clinic for surgical consideration. Due to the morbidity associated with surgical intervention, our primary objective was to investigate the utilization patterns of promotility serotonergic drugs in this population of patients. An additional objective was to explore the morbidity and mortality associated with surgical management in our study population.

## 2. Methods

This study was approved by the institutional research ethics board. The study population was drawn from patients referred to a colorectal motility clinic at a Canadian tertiary care centre from 1996 to 2011. All patients who met inclusion criteria were consecutively sampled. Inclusion criteria were age ≥18, diagnosis of GI motility disorder, and consideration for bowel resection for management of motility disorder. All patients who were later discovered to have a diagnosis of inflammatory bowel disease or a GI motility disorder secondary to spinal cord injury were excluded (Table 1). It was important to exclude other nonmotility disorders or mechanical causes of obstruction. Thus each patient received a work-up including but not limited to upper and lower GI endoscopy, barium swallow, colonic transit studies, and esophageal and anal manometry.

A retrospective cohort study was conducted through retrospective review of prospectively collected clinical data and electronic records. Continuous variables were reported as means with standard deviations. Differences were assessed with the use of independent *t*-tests (continuous data) and Fisher's exact tests (categorical data). Logistic regression analyses were performed to assess whether disease severity or presence of diffuse dysmotility was significantly associated with use of serotonergic drugs. As most patients had abdominal pain, the most objective measure of disease severity prior to bowel resections was the frequency of bowel movements (BMs). This decision was made because most patients had abdominal pain and thus abdominal pain was not felt to be a good metric to stratify disease severity between these patients. Given that frequency of BMs is not normally distributed and tends to be reported as a range, this variable was considered as an ordinal categorical variable. Since promotility serotonergic drugs have been aimed primarily at constipation-dominant patients, these patients were the main focus of our analyses. Diffuse dysmotility was defined as those patients who objectively demonstrated disordered motility in ≥2 parts of their GI tract. An *α* of 0.05 was set as the threshold for rejecting the null hypothesis of no association. All analyses were performed using the SPSS/PASW (v.20) statistical package.

## 3. Results

This study population was comprised of 128 patients with a motility disorder and consisted of both original patients of the senior author and patients referred by others. The majority of these patients (*n* = 125, 97.7%) were women. Only 32 patients of the cohort (25.0%) had ever used serotonergic promotility drugs.

**Figure 1:**
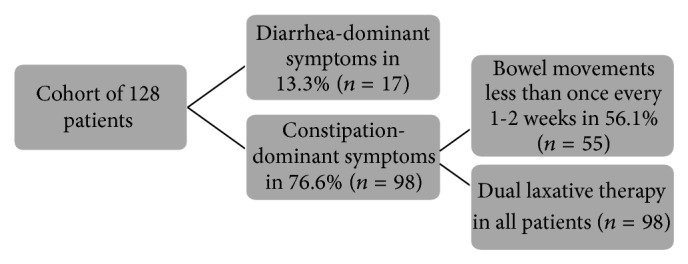
Flow diagram illustrating the percentage of each motility disorder subtype within the cohort. The majority suffered from the constipation-dominant subtype. Of these, over 50% had bowel movements less than once every 1-2 weeks. Dual laxative therapy was used in all constipation-dominant subjects.

**Figure 2:**
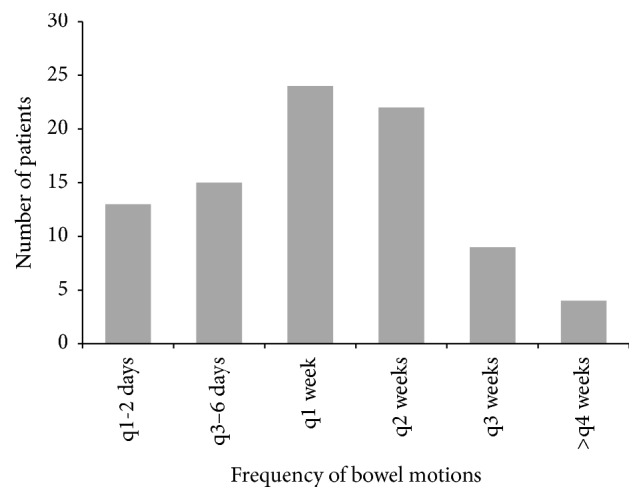
Distribution of disease severity in constipation-dominant patients. Severity is measured in number of bowel motions per duration of time. Disease is said to be severer with greater duration between bowel motions. The majority of patients reported bowel motions once every 1-2 weeks.

The majority of subjects suffered from constipation-dominant symptoms (*n* = 98, 76.6%) (Figures 1 and 2). All constipation-dominant patients used ≥2 laxatives, and the number of BMs reported reflects their bowel habits despite this. All these constipation-dominant patients fit Rome III criteria for constipation-dominant IBS [4].

Most patients in this study population presented with motility disorders that were limited to 1 part of the GI tract (based on preoperative work-up). Approximately 16.4% (*n* = 21) of patients, however, presented with diffuse motility disorders that affected ≥2 parts of their GI tract. This often presented in the form of a patient meeting Rome III criteria for IBS as well as demonstrating evidence of delayed gastric emptying on gastric motility studies [4]. This was defined for this study as diffuse dysmotility and was considered as a dichotomous variable.

On univariable and multivariable analysis (adjusting for presence of diffuse dysmotility), there was no association between the use of serotonergic promotility drugs and severity of constipation (*p* = 0.58 and *p* = 0.41) (Table 1). Of those with diffuse dysmotility, only 9 (42.9%) were known to have tried serotonergic promotility drugs (Table 2). Furthermore, univariable and multivariable (adjusting for constipation severity) analysis showed that the use of promotility serotonergic drugs was not associated with the presence of diffuse dysmotility (*p* = 0.05 and *p* = 0.11) (Table 2). In order to elucidate whether severity of constipation was an effect modifier of diffuse dysmotility in predicting use of promotility serotonergic drugs, an interaction term was created combining these 2 predictors. The interaction term was not significantly associated with use of promotility serotonergic drugs (*p* = 0.52).

**Figure 3:**
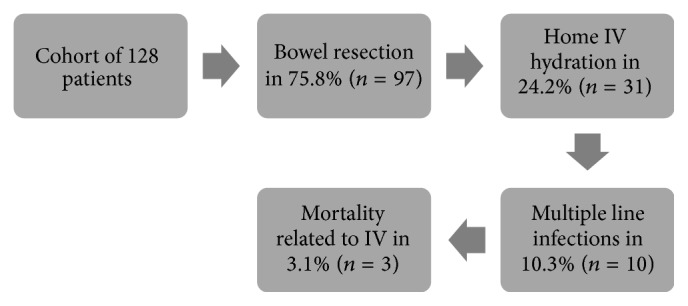
Flow diagram illustrating postsurgical morbidity and mortality associated with intravenous line infections. IV hydration was required in 24.2% (*n* = 31) of patients who underwent surgery. Three patients died as a result of complications of intravenous therapy.

Most patients (*n* = 97, 75.8%) underwent some form of surgical intervention with 24.2% (*n* = 31) subsequently needing home intravenous hydration. Of these, 10 (32.2% of those with home IV hydration and 10.3% of those who had surgery) had multiple line infections and 3 (9.7% of those with home IV hydration and 3.1% of those who had surgery) suffered line-related mortality (2 of line-related septic shock, 1 of air embolism). Of those who underwent surgical resection, 14 (14.4%) required repeat surgery in the form of abdominoperineal resection or permanent ileostomy (Figure 3).

## 4. Discussion

This represents the largest cohort study describing patients with such severe GI motility disorders as to be considered for surgical management. The majority of patients within this cohort suffered from constipation-dominant symptoms and all of these patients were on at least 2 laxatives. Patients were enrolled into this database between 1996 and 2011 and during this time several promotility serotonergic drugs were introduced and/or withdrawn from the Canadian market, including Cisapride and Tegaserod [7, 8]. Thus this cohort of patients was likely impacted by the availability of these drugs.

Of the 128 patients in the study, 97 underwent surgical resection (75.8%) [10]. Of these, 24.2% (*n* = 31) required home IV hydration. Of those that required IV hydration, 10 (32.2%) developed multiple line infections and 3 (9.7%) suffered line-related mortality. This indicates the serious nature of postoperative morbidity and mortality for invasive treatment of refractory constipation.

There is good evidence that serotonergic promotility drugs are effective for treating constipation-dominant IBS. Several randomized studies have concluded that Tegaserod is successful at decreasing symptoms [11–13]. Despite the demonstrated efficacy of serotonergic drugs, our study demonstrated no association between drug use and the severity of constipation (*p* = 0.58). Nor was there an association between serotonergic drug use and presence of diffuse dysmotility on unadjusted and adjusted analyses.

Patients with diffuse dysmotility represent our most difficult patients, the complexity of their management amplified by the absence of one solitary GI tract problem that can be resected. These types of patients especially require the use of all possible medical management prior to considering surgery. In our cohort, management was often initiated at the primary care level and modified upon specialist referral. Despite this, drug utilization appeared to be somewhat random/haphazard. We would expect that patients with diffuse dysmotility and higher constipation severity are more likely to have been prescribed serotonergic drugs. However, our analyses did not show this relationship. This finding may be influenced by the small sample size of our study and the consequent decrease in the robustness of our model.

The NICE and World Gastroenterology Organization's guidelines (2010) both advocate the use of Prucalopride as part of their treatment algorithm [13, 14]. All of our constipation-dominant patients would have qualified under these guidelines. Despite this, 75.8% (*n* = 97) of patients in this cohort underwent surgery with only 25.0% (*n* = 32) of patients having tried a serotonergic drug.

A disparity exists between best available evidence and the actual management of these patients. The likeliest explanation for this is the unavailability of promotility serotonergic drugs in Canada during the therapeutic time frame for these patients. Cisapride was introduced in 1990 and withdrawn in 2000. Tegaserod was introduced in 2002 and withdrawn in 2007. There was an absence of any alternate serotonergic drugs during this time. Primary physicians and specialists therefore likely referred their patients for surgical consideration due to a lack of treatment options. Tegaserod was withdrawn due to a reported increase in incidence of cardiovascular ischemic events in drug users versus placebo (0.11% versus 0.01%, *p* = 0.024). This represents an absolute risk increase of only 0.1% of all (i.e., fatal and nonfatal) CV ischemic events. Furthermore, subsequent analysis of the cardiovascular risk of Tegaserod in multiple studies has questioned the validity of the data that led to its withdrawal [15, 16]. Anderson et al. reported no significant difference in cardiovascular events in a matched cohort study comparing 2603 Tegaserod-treated patients in a 1 : 6 fashion with 15 618 matched patients who were not treated with Tegaserod [15]. Loughlin et al. performed a large matched cohort study using large American health insurance databases and compared 52 229 patients using Tegaserod to 52 229 matched patients not using Tegaserod; they also reported no significant difference in rates of cardiovascular ischemic events [16]. Similar issues surrounded the withdrawal of Cisapride [17]. In contrast, the mortality in our study from line infections alone was 9.7%. Although small subsets of patients who are appropriately screened may experience improvements in the health-related quality of life after surgery [18], several studies have demonstrated the significant morbidity associated with surgical intervention for GI motility disorders [19–21]. A review of 31 series consisting of 310 patients who underwent colectomy for slow transit constipation found a median rate of 18% small bowel obstruction and 14% reoperation [22]. Thus the adverse events reported for serotonergic drug use in this patient population are less likely to have a negative impact than those associated with surgery. These conclusions mirror the WGO and NICE algorithm regarding the use of promotility serotonergic drugs in the treatment of constipation [13, 14]. Furthermore, the economic and psychological burden of major abdominal surgery clearly suggests that an approach utilizing serotonergic drug therapy prior to surgical intervention is preferential in the management of these patients [22, 23].

Currently, if patients fail to respond to Prucalopride, the options for primary physicians are again reduced to the following: (1) applying for special permission to use Tegaserod/Cisapride or (2) proceeding to surgical referral/intervention. Currently regulatory health agencies in North America have allowed Tegaserod to be used in emergency situations, defined as a life-threatening episode or one requiring hospitalization. There are however a number of exclusion criteria, including but not limited to age > 55, obesity, depression, and anxiety [24]. Currently a large number of patients in our cohort would not qualify for Tegaserod therapy. Our cohort of patients did not experience any cardiovascular events during the course of their follow-up periods. The small and inconsistently reported cardiovascular risks of promotility serotonergic drugs must be weighed against the burden of disease as well as the morbidity associated with its surgical management. Certainly, patients with motility disorders who have failed other medical therapy and who also have cardiovascular disease would warrant different considerations.

One major limitation of this study is related to its small and retrospective nature. Though most of the data was prospectively collected, some data points were not collected with this specific study in mind. Another limitation is the self-report nature of some of the drug utilization history; that being said, the majority of drug histories were provided by referring physicians.

## 5. Conclusions

Surgical management of GI motility disorders can result in serious morbidity and mortality. There is evidence that serotonergic promotility drugs may help patients to avoid surgery. Several current guidelines suggest the use of serotonergic drugs before consideration of surgery. Despite this, there was a low and haphazard utilization of these drugs in our cohort. The adverse events reported for serotonergic drugs are less likely to negatively impact this patient population compared to surgical intervention for dysmotility. Future studies that address patients' preferences regarding the balance of risks and benefits are needed, as well as studies that better document drug utilization and quality of life in this population. At the policy level, these findings suggest that the restricted availability of promotility serotonergic drugs such as Tegaserod should be reexamined in populations with severe and refractory GI motility disorders.

## Figures and Tables

**Table 1:** Inclusion & exclusion criteria.

Inclusion criteria	Age ≥ 18
	Diagnosis of GI motility disorder
	Referred for consideration of bowel resection

Exclusion criteria	Inflammatory bowel disease
	Motility disorder secondary to spinal cord injury

**Table 2:** Baseline characteristics.

	Use of serotonergic drugs (*n* = 32)	No serotonergic drugs (*n* = 96)	*p*
Age at symptom onset	1st decade: 29 (90.6%) 2nd decade: 1 (3.1%) 3rd decade: 1 (3.1%) 4th decade: 0 (0.0%) ≥5th decade: 1 (3.1%)	1st decade: 65 (67.7%) 2nd decade: 1 (1.0%) 3rd decade: 5 (5.2%) 4th decade: 8 (8.3%) ≥5th decade: 10 (10.4%)	0.17

Female	32 (100%)	93 (96.9%)	0.57

Constipation-dominant	27 (84.4%)	71 (74.0%)	0.34

Diffuse motility disorder	9 (28.1%)	12 (12.5%)	0.05

Underwent surgery	23 (71.9%)	74 (77.1%)	0.63

## References

[1] Sandler R. S. (1990). Epidemiology of irritable bowel syndrome in the United States. *Gastroenterology*.

[2] Sandler R. S., Everhart J. E., Donowitz M. (2002). The burden of selected digestive diseases in the United States. *Gastroenterology*.

[3] Spiegel B. M. R. (2009). The burden of IBS: looking at metrics. *Current Gastroenterology Reports*.

[4] Drossman D. A. (2007). Introduction. The Rome Foundation and Rome III. *Neurogastroenterology & Motility*.

[5] Pemberton J. H., Rath D. M., Ilstrup D. M. (1991). Evaluation and surgical treatment of severe chronic constipation. *Annals of Surgery*.

[6] FitzHarris G. P., Garcia-Aguilar J., Parker S. C. (2003). Quality of life after subtotal colectomy for slow-transit constipation: both quality and quantity count. *Diseases of the Colon and Rectum*.

[7] Smalley W., Shatin D., Wysowski D. K. (2000). Contraindicated use of cisapride: impact of food and drug administration regulatory action. *The Journal of the American Medical Association*.

[8] US Food and Drug Administration FDA Announces Discontinued Marketing of GI Drug, Zelnorm, for Safety Reasons. http://www.fda.gov/NewsEvents/Newsroom/PressAnnouncements/2007/ucm108879.htm.

[9] Tack J., Müller-Lissner S., Bytzer P. (2005). A randomised controlled trial assessing the efficacy and safety of repeated tegaserod therapy in women with irritable bowel syndrome with constipation. *Gut*.

[10] Kidane B., Lam J., Manji F., Gupta V., Chadi S. A., Taylor B. M. (2015). Histological findings in resected bowel of motility-disordered patients. *American Surgeon*.

[11] Kellow J., Lee O. Y., Chang F. Y. (2003). An Asia-Pacific, double blind, placebo controlled, randomised study to evaluate the efficacy, safety, and tolerability of tegaserod in patients with irritable bowel syndrome. *Gut*.

[12] Nyhlin H., Bang C., Elsborg L. (2004). A double-blind, placebo-controlled, randomized study to evaluate the efficacy, safety and tolerability of tegaserod in patients with irritable bowel syndrome. *Scandinavian Journal of Gastroenterology*.

[13] World Gastroenterology Organisation (2010). *World Gastroenterology Organisation Global Guidelines. Constipation: A Global Perspective*.

[14] National Institute for Health and Clinical Excellence (2010). *NICE Technology Appraisal Guidance (TA211). Prucalopride for the treatment of Chronic Constipation in Women*.

[15] Anderson J. L., May H. T., Bair T. L., Muhlestein J. B., Horne B. D., Carlquist J. F. (2009). Lack of association of tegaserod with adverse cardiovascular outcomes in a matched case-control study. *Journal of Cardiovascular Pharmacology and Therapeutics*.

[16] Loughlin J., Quinn S., Rivero E. (2010). Tegaserod and the risk of cardiovascular ischemic events: an observational cohort study. *Journal of Cardiovascular Pharmacology and Therapeutics*.

[17] Wysowski D., Corken A., Gallo-Torres H. (2001). Postmarketing reports of QT prolongation and ventricular arrhythmia in association with cisapride and food and drug administration regulatory actions. *The American Journal of Gastroenterology*.

[18] Lam J. Y., Kidane B., Manji F., Taylor B. M. (2015). Improved health-related quality of life after surgical management of severe refractory constipation-dominant irritable bowel syndrome. *International Surgery*.

[19] Nyam D. C. N. K., Pemberton J. H., Ilstrup D. M., Rath D. M. (1997). Long-term results of surgery for chronic constipation. *Diseases of the Colon and Rectum*.

[20] Knowles C. H., Scott M., Lunniss P. J. (1999). Outcome of colectomy for slow transit constipation. *Annals of Surgery*.

[21] Beck D. E., Opelka F. G., Bailey H. R., Rauh S. M., Pashos C. L. (1999). Incidence of small-bowel obstruction and adhesiolysis after open colorectal and general surgery. *Diseases of the Colon and Rectum*.

[22] Chapman C. R., Cox G. B. (1977). Anxiety, pain, and depression surrounding elective surgery: a multivariate comparison of abdominal surgery patients with kidney donors and recipients. *Journal of Psychosomatic Research*.

[23] US Food and Drug Administration (2012). *Zelnorm (Tegaserod Maleate) Information*.

[24] Health Canada (2007). *Voluntary Suspension of Sales of Zelnorm due to Cardiovascular Ischemic Events*.

